# A covalent organic framework-based route to the *in situ* encapsulation of metal nanoparticles in N-rich hollow carbon spheres†
†Electronic supplementary information (ESI) available: Experimental details and catalysts characterization. See DOI: 10.1039/c6sc01659f


**DOI:** 10.1039/c6sc01659f

**Published:** 2016-05-31

**Authors:** Liyu Chen, Lei Zhang, Zhijie Chen, Hongli Liu, Rafael Luque, Yingwei Li

**Affiliations:** a Key Laboratory of Fuel Cell Technology of Guangdong Province , School of Chemistry and Chemical Engineering , South China University of Technology , Guangzhou 510640 , China . Email: liyw@scut.edu.cn; b Departamento de Química Orgánica , Universidad de Córdoba , Edif. Marie Curie, Ctra Nnal IV-A, Km 396 , E14014 , Córdoba , Spain . Email: q62alsor@uco.es

## Abstract

Metal nanoparticles (NPs) encapsulated in hollow nanostructures hold great promise for a variety of applications. Herein, we demonstrate a new concept where covalent organic frameworks (COFs) doped with metal cations can be readily used as novel precursors for the *in situ* encapsulation of metal NPs into N doped hollow carbon spheres (NHCS) through a controlled carbonization process. The obtained Pd@NHCS composites show a significantly enhanced catalytic activity and selectivity in the hydrogenation of nitrobenzene in ethanol and oxidation of cinnamyl alcohol compared with that of the conventional Pd/N–C and commercial Pd/C catalysts. The excellent catalytic performance should be related to the synergism of the porous hollow spheric structure, highly dispersed Pd NPs, and uniform distribution of N dopants on the materials. We believe that this newly developed methodology could be extended to the synthesis of other metal NPs@NHCS composites for a variety of advanced applications.

## Abstract 2

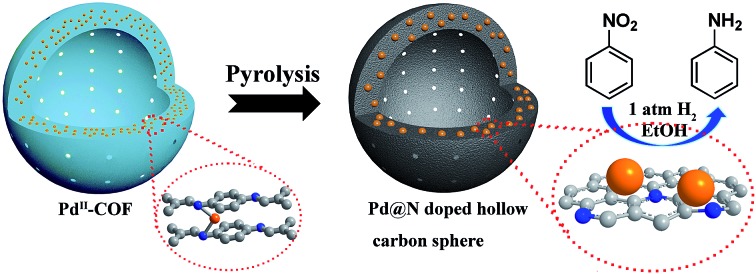
Covalent organic frameworks doped with metal cations can be used as novel precursors for the *in situ* encapsulation of metal NPs into N doped hollow carbon spheres. The integration of the hollow structure, N dopant and ultrafine Pd NPs gives the hybrid nanocomposites advanced catalytic performance.

## Introduction

Hollow-structured porous materials, with large internal voids and porous shells, have promising applications in a variety of fields, such as gene therapy, confined catalysis, adsorption and storage.^1^ Among such systems, hollow carbon spheres (HCS) are very attractive owing to their outstanding features, such as their low density, good thermal stability, and high permeability.^2^ Recently, considerable interest has been raised for employing HCS as nanoreactors by encapsulating catalytically active metal nanoparticles (NPs) in the porous shell of HCS.^3^ The shell could function as a barrier to prevent the encapsulated metal NPs from coalescing, and the pore in the shell connecting the hollow core can provide a highway network for the free diffusion of reactant molecules to access the metal active sites.^4^ Moreover, it has been demonstrated that the incorporation of heteroatoms (*e.g.*, nitrogen, boron, sulphur and phosphorus) into the carbon lattice can significantly improve its chemical and electrical properties, and also enhance the interactions between the carbon support and the embedded metal NPs to trigger superior catalytic performance.^5^

Considerable efforts have been devoted to exploring effective strategies for the preparation of metal NPs encapsulated within N doped HCS (NHCS).^6^ Typically, the synthesis requires the employment of an easily removable material (*e.g.*, SiO_2_) as the hard template for the coating and subsequent pyrolysis of nitrogen containing precursors (*e.g.*, dopamine and resorcinol–formaldehyde) to afford a N-rich carbon shell.^7^ Then NHCS could be obtained after the removal of siliceous components by treatment with HF solution, which is then used as the support for the immobilization of metal NPs by impregnation or coprecipitation. Although this traditional synthetic route has been widely used, it involves tedious and complicated steps, and requires hazardous reagents (*i.e.*, HF) for the removal of hard templates and excess reducing reagents for the reduction of metal precursors. Moreover, the postloading of metal NPs to the support would lead to insufficient attachment of the NPs to the support.3*c* Therefore, it is highly desirable to develop a simple and efficient strategy to integrate metal NPs into NHCS for the design of advanced nanocatalysts.

Recently, solid-state pyrolysis of porous organic networks, such as metal-organic frameworks (MOFs)^8^ and covalent organic frameworks (COFs),^9^ has emerged as a simple, efficient and promising approach for the fabrication of nanostructured materials. Especially, COFs are only composed of organic skeletons, making them outstanding templates for the facile preparation of porous carbons.^10^ By carefully choosing construction units, COFs with various morphographies and functions could be synthesized, thus allowing control of the shape and composite of the final nanostructured materials *via* thermolysis.^11^ Nevertheless, to the best of our knowledge, the employment of COF-based materials as pyrolysis sacrificial precursors for NHCS supported metal NPs composites has never been explored.

**Scheme 1:**
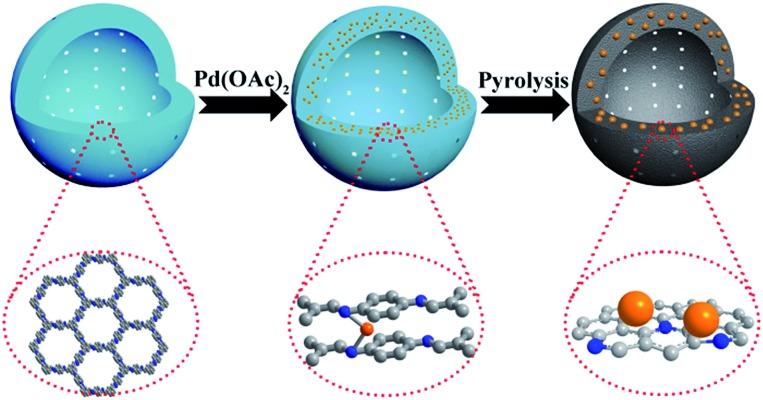
Schematic illustration of the fabrication of Pd@NHCS. Color coding: grey, carbon; blue, nitrogen; orange, Pd precursors or NPs.

In this work, we report a novel one-step synthesis strategy for the *in situ* fabrication of NHCS encapsulated metal NPs by using metal ion doped COF as a precursor. As a proof of concept, we chose LZU1,^12^ a hollow spheric COF, as the NHCS template. In this COF, the eclipsed nitrogen atoms in adjacent layers can serve as the coordination sites for transition cations such as Pd^2+^ (Scheme 1). During the annealing process under an inert atmosphere, Pd cations will be reduced to form Pd NPs, meanwhile the N-containing organic linkers will serve as both the carbon and nitrogen sources for the formation of NHCS. Through this rational design, the high nitrogen doping in the NHCS support could geometrically and electronically modify the *in situ* growth of Pd NPs and the hollow structure could facilitate the diffusion of the substrate, endowing the obtained material with a superior catalytic capability. As demonstrated in the hydrogenation of nitrobenzene and oxidation of cinnamyl alcohol, this unique Pd@NHCS hybrid material exhibits significantly improved catalytic performances in terms of activity and selectivity as compared to the Pd/N–C and commercial Pd/C catalysts under identical conditions.

## Results and discussion

LZU1 was synthesized following a previously reported protocol with slight modifications.^12^ Pd(ii)-doped LZU1 (Pd^II^-LUZ1) was facilely prepared through a simple impregnation of LZU1 with palladium acetate. Field emission scanning electron microscopy (FESEM) and transmission electron microscope (TEM) images show that Pd^II^-LZU1 is mainly composed of hollow spheres with a diameter of about 250 nm and a shell thickness of *ca.* 40 nm (Fig. S1a† and 1a and b). X-ray photoelectron spectroscopy (XPS) measurements were performed to investigate the intermolecular interaction between the COF and Pd(OAc)_2_. For the Pd 3d5/2 spectrum, one peak at 337.8 eV was observed for Pd^II^-LZU1, characteristic of divalent Pd ions (Fig. S2†). This value shifts negatively in comparison with that of free Pd(OAc)_2_.^13^ In addition, the parent LZU1 contains only one kind of N, *i.e.*, Schiff base nitrogen (Fig. 2a). After the immobilization of Pd(OAc)_2_, a new N 1s peak at *ca.* 400 eV was observed. The shift of the N 1s peak toward a higher binding energy reflects a decrease in the electron density of N. These results indicate that there exists a strong coordination between Pd(OAc)_2_ and the imine groups of LZU1.

**Fig. 1:**
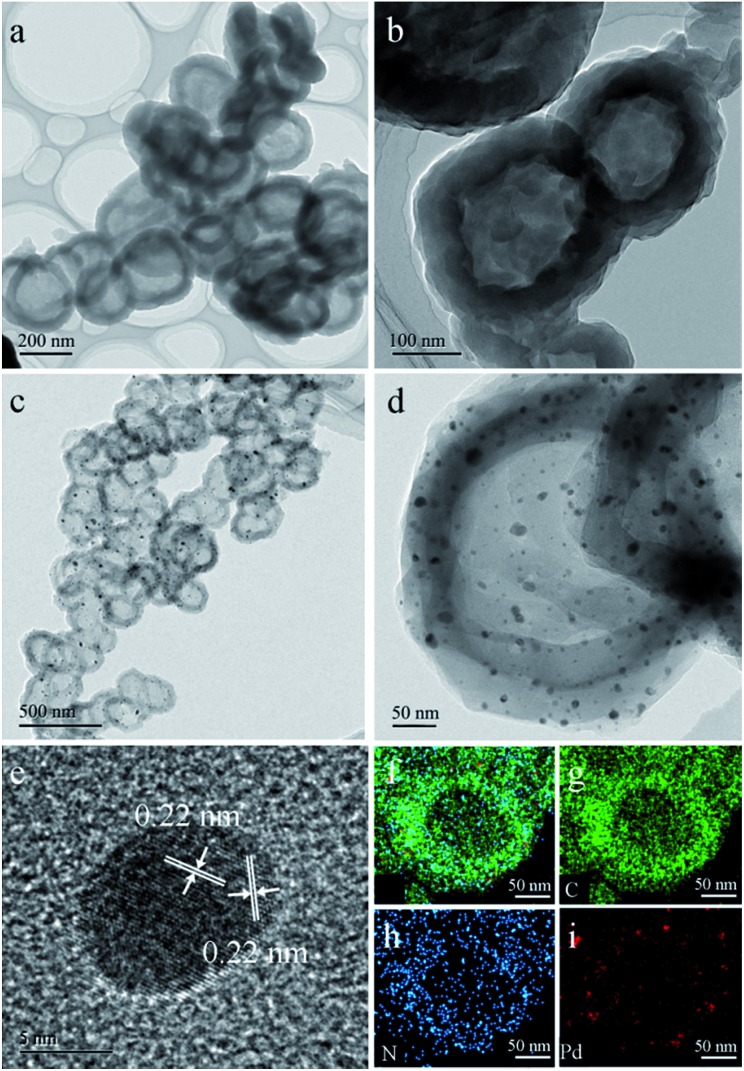
(a and b) TEM images of Pd^II^-LUZ1. (c and d) TEM images of Pd@NHCS(500). (e) HRTEM image of Pd NPs. (f–i) EDS elemental mapping of a Pd@NHCS(500) particle (green: C; blue: N and red: Pd).

**Fig. 2:**
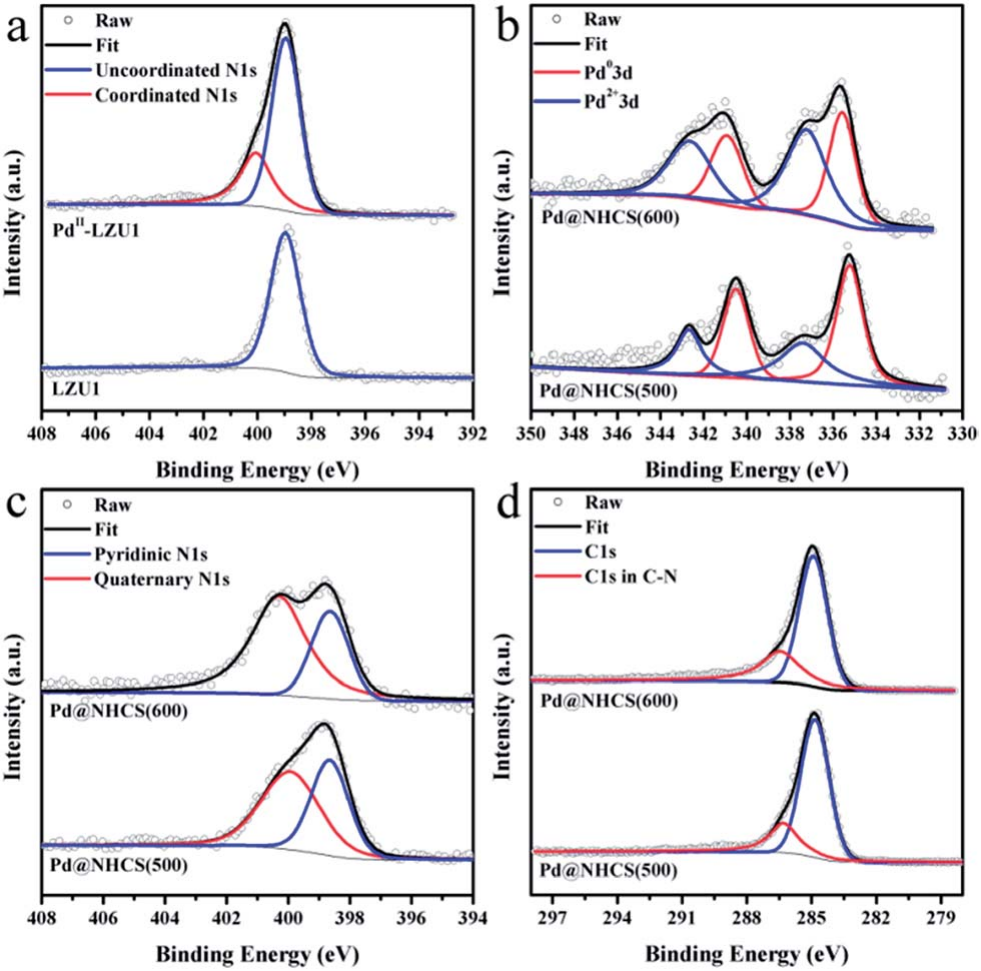
(a) XPS N 1s of LUZ1 before and after the immobilization of Pd(OAc)_2_. High-resolution (b) Pd 3d spectra, (c) N 1s spectra, and (d) C 1s spectra for Pd@NHCS(500) and Pd@NHCS(600).

**Fig. 3:**
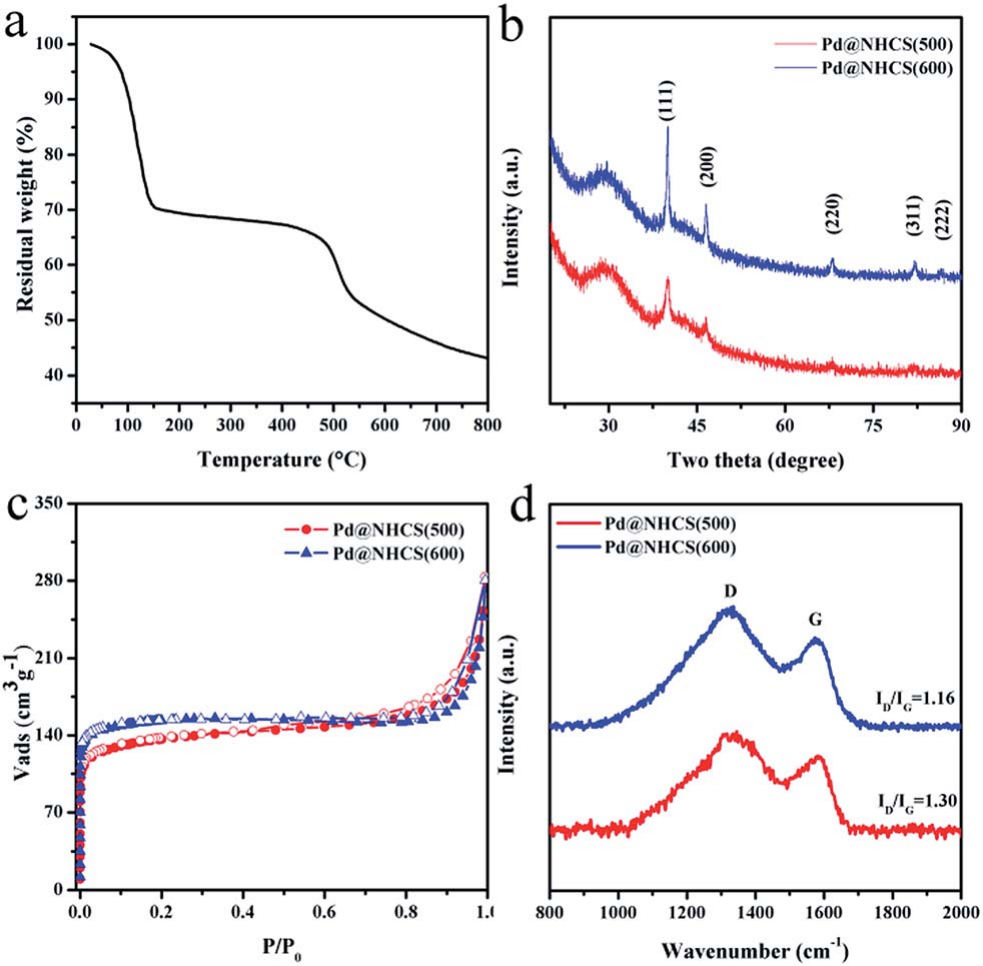
(a) TGA curve of Pd^II^-LUZ1. (b) Powder XRD patterns for Pd@NHCS(500) and Pd@NHCS(600). (c) Nitrogen adsorption isotherms for Pd@NHCS(500) and Pd@NHCS(600). (d) Raman spectra for Pd@NHCS(500) and Pd@NHCS(600).

As observed from the thermogravimetric analysis (TGA) curves (Fig. 3a), Pd^II^-LZU1 begins to decompose when the temperature is increased to *ca.* 500 °C under argon. Therefore, the Pd^II^-LZU1 composites were annealed at 500 °C for 3 h with a heating rate of 1 °C min^–1^ to produce the final composite, which is denoted as Pd@NHCS(500). The carbonized products show a similar morphology compared to the precursor (Fig. S1b†). The diameter of the spheres and the thickness of the shell measured from the TEM images are *ca.* 200 nm and 30 nm, respectively (Fig. 1c and d). The observed smaller particles in comparison with the precursors are reasonable, as a consequence of the volume shrinking after the high-temperature heating. The corresponding dark field scanning transmission electron microscopy (DFSTEM) images (Fig. S3†) clearly show hollow shells. The Pd NPs are uniformly distributed with an average size of 6 nm, and mostly located within the carbon shell. A representative high-resolution TEM image of Pd-in-UiO-67 displays distinct lattice fringes with *d* spacings of 0.22 nm, corresponding to the (111) lattice spacing of face-centered cubic Pd (Fig. 1e). The surface of Pd is not coated by carbon layers, indicating that the Pd NPs are embedded within the pores or supported on the surface of NHCS, and are thus accessible to the reactants. The energy dispersive spectrometer (EDS) mapping images (Fig. 1f–i) reveal the uniform distribution of C, N and Pd elements all over the support. The Pd content in the Pd@NHCS(500) is 2.4 wt%, as measured by atomic absorption spectroscopy (AAS). The elemental analysis results further reveal that Pd@NHCS(500) is composed of C, H and N elements, and the nitrogen content is as high as 11.0 wt% (Table S1†).

When further increasing the annealing temperature to 600 °C, some particles with a broken outer shell were observed (Fig. S4†). The as-formed Pd NPs show some aggregation, with an average size of 19 nm. Elevating the annealing temperatures also leads to a decrease in nitrogen content and an increase in the Pd content of the resultant samples (Table S1†).

The crystalline phases and structures of the materials were examined by powder X-ray diffraction (XRD) and Raman analysis. The characteristic XRD peaks (Fig. 3b) at 40.1, 46.7, 68.2, 82.2 and 86.7 may be assigned to the (111), (200), (220), (311) and (222) reflections of Pd (JCPDS 65-6174), respectively. No peaks for other possible impurities were detected, indicating the high purity of the synthesized materials. Increasing the calcination temperature enhances the intensity of the Pd diffraction peak, suggesting a bigger size and higher crystallinity degree of Pd NPs as the calcination temperature increases, which is in good agreement with that observed in the TEM analysis. A broad and weak XRD peak around 29.5° was detected (Fig. 3b), confirming the existence of amorphous carbon. In addition, the Raman spectra show two broad bands (Fig. 3d), in which the G band at ∼1584 cm^–1^ indicates the in-plane vibration of the aromatic carbon atoms, while the D band at ∼1313 cm^–1^ is a disorder induced Raman feature caused by the non-perfect crystalline structure.^14^ The relative ratio of D band to G band (*I*_D_/*I*_G_) in the Raman spectra of Pd@NHCS(500) and Pd@NHCS(600) is 1.30 and 1.16, respectively. The D band appears to be stronger than the G band, implying the generation of large amounts of defects because of the high nitrogen doping percentage in the resulting composites.

N_2_ adsorption–desorption experiments were performed at 77 K to measure the surface area and porosity of the resultant NHCS supported Pd composites. The adsorption/desorption isotherms are shown in Fig. 3c. Pd@NHCS samples show a typical adsorption curve of type I plus IV with steep increases at low relative pressures and an obvious hysteresis loop in the *P*/*P*_0_ range of 0.8–1, revealing the presence of micro-, meso- and macropores. These Pd@NHCS materials have Brunauer–Emmett–Teller (BET) surface areas of 450–550 m^2^ g^–1^ and pore volumes of 0.19–0.24 cm^3^ g^–1^ (Table S2†). The porous structure of the NHCS supports would benefit the transportation of molecules to the embedded Pd NPs in catalytic transformations.

XPS measurements were conducted to investigate the electronic and structural properties of Pd@NHCS composites. As illustrated in Fig. 2b, Pd@NHCS(500) contains *ca.* 60% Pd^0^, as revealed by the Pd 3d5/2 XPS peaks at 335.2 and 337.4 eV; a relatively lower percentage of Pd^0^ (*ca.* 43%) was observed for Pd@NHCS(600). The N 1s spectra of the Pd@NHCS composites can be deconvoluted into two peaks at 398.6 and 399.9 eV, respectively, which are consistent with the pyridinic N and quaternary N in the carbon texture (Fig. 2c). The ratio of graphitic nitrogen to pyridinic nitrogen in Pd@NHCS is increased when the pyrolysis temperature is enhanced (*e.g.*, 1.3 for 500 °C and 2.2 for 600 °C), implying a higher graphitization degree at elevated temperatures. The C 1s XPS spectra of the Pd@NHCS composites presented in Fig. 2d can be deconvoluted into two major peaks. The peak at 284.6 eV corresponds to the sp^2^ graphitic carbon species, while the other one at 285.4 eV may be assigned to the sp^2^-carbon containing nitrogen atoms, suggesting the presence of nitrogen functionalities on the surface of NHCS.

**Fig. 4:**
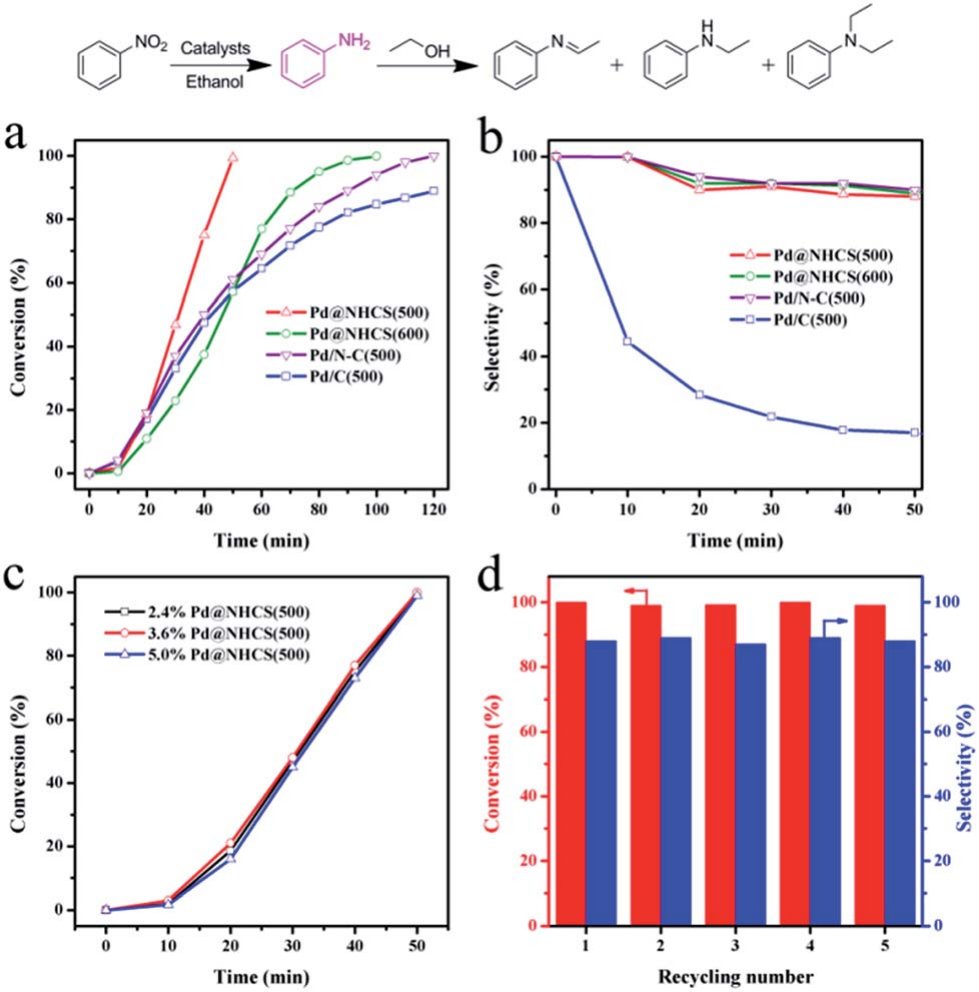
(a) Nitrobenzene conversions and (b) selectivities to aniline *versus* time in the hydrogenation of nitrobenzene over Pd@NHCS(500), Pd@NHCS(600), Pd/C–N(500) and Pd/C(500). (c) Effect of Pd loading on the catalytic performance of Pd@NHCS(500). (d) Recyclability of 2.4% Pd@NHCS(500) in the hydrogenation of nitrobenzene.

The catalytic efficiencies of the as-synthesized nanohybrid materials were evaluated using the hydrogenation of nitrobenzene to aniline as a model reaction.^15^ The reactions were performed under an atmospheric pressure of H_2_ and room temperature (25 °C), and the reaction profiles are shown in Fig. 4. It was found that the hydrogenation of nitrobenzene in alcohol could afford not only aniline, but also an imine, *N*-ethylaniline and tertiary amine as by-products coming from the further *N*-alkylation of amine with alcohol.^16^ Pure NHCS(500) gave essentially no conversion under the reaction conditions, implying the need of a metal to perform the hydrogenation of nitrobenzene. Pd@NHCS(500) showed a very high activity, with a complete conversion of nitrobenzene and 88% aniline selectivity within only 50 min. Pd@NHCS(600) was less active than Pd@NHCS(500), requiring a much longer time (*ca.* 100 min) to obtain a quantitative conversion of nitrobenzene. The relatively poor performance may result from the larger particle size of the Pd NPs in Pd@NHCS(600) (as demonstrated by the TEM analysis). Nevertheless, the Pd@NHCS(600) catalyst could also achieve a similarly high selectivity to aniline as Pd@NHCS(500) (Fig. 4b).

To illustrate the benefit of the hollow spheric structure and N dopant to the catalytic performance, we also prepared Pd NPs supported on N doped carbon and commercial carbon materials for comparison. First, N doped carbon was prepared according to our previous report.^17^ The N–C material was then impregnated with Pd(OAc)_2_ and treated at 500 °C for 3 h under argon. The resulting material is denoted as Pd/N–C(500). The TEM image shows that Pd/N–C(500) has a comparable average size of Pd NPs to that of Pd@NHCS(500) (Fig. S5a†). AAS analysis demonstrated that Pd/N–C(500) had a similar Pd loading (2.1 wt%) to that of Pd@NHCS(500). In the hydrogenation of nitrobenzene, Pd/N–C(500) exhibited a moderate activity and produced aniline in 89% yield in 120 min. The substantially lower catalytic activity for Pd/N–C(500), as compared to Pd@NHCS(500), indicates that the hollow structure and porous shell of the NHCS may help the substrate to access Pd NPs, thus showing an enhanced reactivity.

On the other hand, Pd(OAc)_2_ was impregnated onto commercial carbon followed by annealing at 500 °C for 3 h to afford Pd/C(500). Under similar reaction conditions, Pd/C(500) showed the worst activity, giving only a 89% conversion in 120 min. Interestingly, Pd/C(500) exhibited a different product distribution compared with those of Pd@NHCS and Pd/N–C. As shown in Table S3,† the selectivity to aniline was very low (12%), while the mono- or di-*N*-alkylation product became the main product over the Pd/C(500) catalyst. To gain more in-depth information about the different catalytic behaviors, the Pd@NHCS(500) and Pd/C(500) materials were characterized by TEM, AAS and XPS. The as-prepared Pd NPs in Pd/C(500) have a similar size distribution, Pd loading (2.0 wt%) and percentage of Pd^0^ as that of Pd@NHCS(500) (Fig. S5b and S6†). However, interestingly, the binding energy of Pd 3d is shifted to a higher value by approximately 0.5 eV. This could be due to the lack of an electron donating dopant (*e.g.*, N) in the carbon support, resulting in more positively charged Pd NPs on Pd/C(500).

*In situ* attenuated total reflection infrared (ATR-IR) spectroscopy was further employed to gain some molecular insights into the surface adsorption property of the catalysts for ethanol. Initially, N_2_-saturated ethanol adsorption on different catalyst surfaces was investigated. The difference spectra of adsorbed ethanol on Pd@NHCS(500) is displayed in Fig. S7.† In this plot, the spectrum of the pristine Pd@NHCS(500) (Fig. S8†) was subtracted. Bands located in the region of 1200–1500 cm^–1^ are characteristic of C–H stretches for methyl and methylene groups in ethanol.^18^ These bands show no obvious change in intensity with time, implying a weak absorption of ethanol on the Pd@NHCS(500) material. However, for Pd/C(500), these bands gradually become weaker with time, suggesting that ethanol might adsorb strongly on Pd/C(500). Nitrogen functionalities on NHCS could act as coordination donor ligands to tune the electronic properties of Pd NPs to exhibit a comparatively higher negative charge to that of Pd/C(500) (Fig. S6†). Considering alcohols may be adsorbed on the Pd surface *via* the lone electron pair of the oxygen atom, the electron-enriched Pd in Pd@NHCS(500) may depress the adsorption of ethanol while positively charged Pd in Pd/C(500) facilitates this adsorption.^19^ Therefore, it is reasonable that ethanol could undergo *N*-alkylation with aniline when using Pd/C(500) as a catalyst, while the alkylation reaction is suppressed over the Pd@NHCS(500) catalyst.

The proposed protocol could be applied for the preparation of Pd@NHCS with different Pd loadings, simply by changing the impregnated amounts of Pd(OAc)_2_ on COFs. As measured by AAS, we could achieve different Pd loadings (*e.g.*, 2.4%, 3.6% and 5.0%) on NHCS. As shown in Fig. S9,† these samples showed a comparable size distribution of Pd NPs. These composites show similar catalytic activities for nitrobenzene hydrogenation when the same amount of Pd (1 mol% to substrate) was used, indicating that the loading of Pd on NHCS would not play a critical role in the activity (Fig. 4c).

The stability and reusability are of great importance for the practical application of a heterogeneous catalyst. After the reaction, the catalyst was separated by centrifugation. AAS analysis of the reaction solution showed that the concentration of palladium in the solution was below the detection limit, suggesting that leaching of active Pd during the reaction was negligible. The used catalyst was thoroughly washed with ethanol and then dried under vacuum to remove the residual solvent. The results shown in Fig. 4d reveal that Pd@NHCS(500) could be regenerated and reused at least five times in subsequent reactions without a significant loss of catalytic activity and selectivity. The metal content of the reused catalysts are almost the same as the fresh one, as determined by AAS. TEM images also show that no apparent Pd aggregation could be observed on the recycled catalysts (Fig. S10†). These results demonstrate that the highly active Pd@NHCS(500) catalyst is stable and reusable under the investigated conditions.

Encouraged by these promising results, we further examined the catalytic activity of these Pd catalysts for the aerobic oxidation of cinnamyl alcohol. The reactions were carried out at 80 °C under air atmosphere and base-free conditions. As shown in Table S4,† compared with Pd/C–N(500) and Pd/C(500), Pd@NHCS(500) exhibited the highest catalytic activity, which provided a quantitative conversion of cinnamyl alcohol into cinnamyl aldehyde within 10 h. These results further support the conclusion that porous hollow spheric structures, highly dispersed Pd NPs and a uniform distribution of N dopants contribute to achieving superior catalytic activities.

## Conclusions

In summary, we have demonstrated a novel and facile *in situ* strategy to synthesize metal NPs encapsulated into the shell of nitrogen doped hollow carbon spheres (NHCS) by using metal ions doped COFs as the precursors through a simple annealing process. The obtained Pd@NHCS composites exhibit unprecedented catalytic performances, in terms of activity and selectivity, that are superior to conventional Pd/N–C and commercial Pd/C catalysts in the hydrogenation of nitrobenzene and oxidation of cinnamyl alcohol. The obtained excellent catalytic properties originated from the synergism of the porous hollow spheric structure, highly dispersed Pd NPs, and uniform distribution of N dopants. This proposed protocol can simplify the synthesis process, with the elimination of hard templates and a significant reduction of waste, as compared to other conventional methodologies and might be extended to the preparation of other metal NPs@NHCS composites for potential applications in a variety of fields including heterogeneous catalysis, as demonstrated here.

## Supplementary Material

Supplementary information
