# Supplementary material for: A covalent organic framework-based route to the in situ encapsulation of metal nanoparticles in N-rich hollow carbon spheres
Source: Chem Sci. 2016 May 31;7(9):6015–20. doi: 10.1039/c6sc01659f (PMC6022137; doi:10.1039/c6sc01659f)
Supplement: Supplementary file 1 [file SC-007-C6SC01659F-s001.pdf]

Electronic Supplementary Material (ESI) for Chemical Science.
This journal is © The Royal Society of Chemistry 2016

Supporting Information

A covalent organic framework-based route to in-situ encapsulation of metal nanoparticles in N-rich hollow carbon spheres

Liyu Chen,[a] *Lei Zhang,*[a] *Zhijie Chen,*[a] *Hongli Liu,*[a] *Rafael Luque,**[b] *and Yingwei Li**[a]

[a] Key Laboratory of Fuel Cell Technology of Guangdong Province, School of Chemistry and Chemical Engineering, South China University of Technology, Guangzhou 510640, China.

[b] Departamento de Química Orgánica, Universidad de Córdoba, Edif. Marie Curie, Ctra Nnal IV-A, Km 396, E14014, Córdoba (Spain)

* Corresponding authors. Email: liyw@scut.edu.cn, q62alsor@uco.es

Experimental

1. Catalysts preparation

All chemicals were purchased from commercial sources and used without further treatments.

1.1 Synthesis of LZU1

1,3,5-triformylbenzene (48 mg) and 1,4-diaminobenzene (48 mg) were dissolved in 3 mL of 1,4-dioxane. The mixture was then transferred into a 25 mL tube. After adding 0.6 mL of 3.0 mol/L aqueous acetic acid, the mixture was flash frozen using a liquid-nitrogen bath. The tube was then sealed after degassing for 15 min. Upon warming to room temperature, the tube was heated at 120 °C for 3 days. The produced products were isolated by centrifugation, washed with DMF (30 mL × 3), THF (30 mL × 3), and dichloromethane (30 mL × 3) successively. The obtained solids were dried under vacuum at 100 °C for 10 h.

1.2 Synthesis of Pd^{II}-LUZ1

LZU1 (80 mg) and palladium acetate (3.1 mg) were dispersed in 10 mL dichloromethane in a 25 mL flask. Then the mixture was kept stirring for 24 h at room temperature. The products were collected via centrifugation, washed with dichloromethane (3 x 20 mL). Finally, the sample was dried in air at room temperature for 1 h.

1.3 Synthesis of Pd@NHCS

The prepared Pd^{II}-LUZ1 was carbonized at different temperatures under argon atmosphere (constant flow at 40 mL min^{-1}) in a tubular furnace. The temperature was

first heated to 150 °C at a heating rate of 1 °C min^{-1} and was held at this temperature for 2 h. The temperature was then raised to a particular value at a rate of 1 °C min^{-1} and was maintained at the final temperature for 3 h. The prepared catalyst was denoted as Pd@NHCS(X), where X indicated the pyrolysis temperature.

1.4 Synthesis of Pd/N-C(500)

The N-C support was synthesized by direct pyrolysis of ZIF-67, followed by removing the Co component in aqua regia for 24 h. Next, the N-C support was isolated by centrifugation and washed several times with deionized water and absolute ethanol before drying under vacuum at 150 °C overnight. Pd/N-C(500) was obtained by impregnating N-C (100 mg) with palladium acetate (8.4 mg) in dichloromethane (10 mL). The solid was treated at 500 °C under argon atmosphere following the same procedures for the preparation of Pd@NHCS(500).

1.5 Synthesis of Pd/C(500)

Pd/C(500) was obtained following the same procedures as for Pd/N-C(500) except using activated carbon as support.

2. Catalyst characterization and catalytic reactions

2.1 Characterization

The BET surface area measurements were performed with N_2 adsorption isotherms at 77 K on a Micromeritics ASAP 2020M instrument. Before the analysis, the samples were evacuated at 100 °C for 2 h. Powder X-ray diffraction patterns of the samples were recorded on a Rigaku diffractormeter (D/MAX-IIIA, 3 kW) employing Cu Kα radiation (λ = 0.1543 nm) at 40 kV, 40 mA at room temperature. TGA of Pd^{II}-

LZU1 was performed on a NETZSCH STA449C under argon atmosphere. X-ray photoelectron spectroscopy (XPS) measurements were performed on on a Kratos Axis Ultra DLD system with a base pressure of 10^{-9} Torr. The size and morphology of samples were investigated by using a transmission electron microscope (JEM-2100F) with EDX analysis (XFlash 5030T) operated at 200 kV. The palladium contents of the samples were determined quantitatively by atomic absorption spectroscopy (AAS) on a HITACHI Z-2300 instrument.

2.2 Catalytic reactions

Typical procedures for hydrogenation of nitrobenzene: Nitrobenzene (0.1 mmol) and 1 mol% Pd catalyst (4.4 mg, 5.1 mg and 5.3 mg for Pd@NHCS(500), Pd/C-N(500) and Pd/C(500), respectively) were added to 2 mL of ethanol. The reaction mixture was stirred at room temperature under 1 atm hydrogen atmosphere. Upon reaction completion, the catalyst particles were removed from the solution by filtration and washed with ethanol. The liquid phase was subsequently analyzed by GC/MS (Agilent Technologies 7890B-5977A equipped with a 0.25 mm × 30 m HP-5MS capillary column).

For the recyclability tests, nitrobenzene (0.1 mmol) and recovered catalyst (4.4 mg) were added to 2 mL of ethanol. The reaction mixture was stirred at room temperature under 1 atm hydrogen atmosphere. Each time, the catalyst was isolated from the reaction solution at the end of the reaction, washed with ethanol, and then dried at 150 °C under vacuum.

Typical procedures for aerobic oxidation of cinnamyl alcohol: cinnamyl alcohol

(0.1 mmol) and 1 mol% Pd catalyst (4.4 mg, 5.1 mg and 5.3 mg for Pd@NHCS(500), Pd/C-N(500) and Pd/C(500), respectively) were added to 2 mL of toluene. The reaction mixture was stirred at 80 °C under air atmosphere. Upon reaction completion, the catalyst particles were removed from the solution by filtration and washed with toluene. The liquid phase was subsequently analyzed by GC/MS (Agilent Technologies 7890B-5977A equipped with a 0.25 mm × 30 m HP-5MS capillary column).

2.3 In Situ ATR-IR Spectroscopy

ATR-IR spectra were recorded on a Thermo Fisher iS10 equipped with a liquid nitrogen cooled MCT detector. The spectra were obtained by averaging 32 scans at a resolution of 1 cm^{-1}. The thin film of catalyst powder deposited on the ZnSe element for ATR-IR spectroscopic study was prepared as follow. A suspension of ca. 50 mg of catalyst powder in 2 mL of chloroform was placed in an ultrasonic bath for 1 h in order to obtain a uniform suspension. A thin layer of solution was spread onto a ZnSe internal reflection element (IRE) and dried out at room temperature. This procedure was repeated six times, subsequently the sample was dried in a vacuum oven for a complete evaporation of methanol.

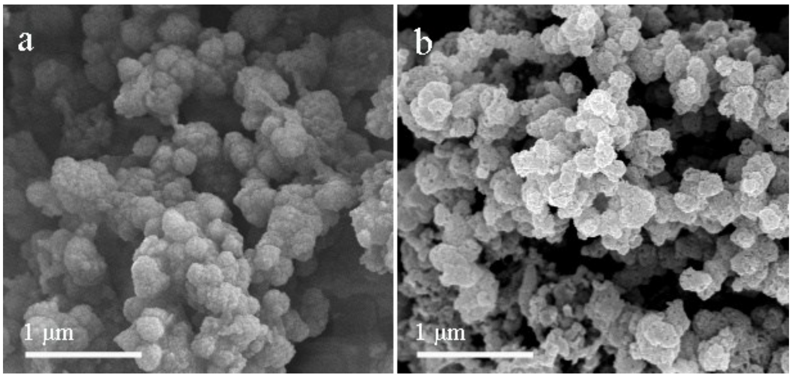

Figure S1. FESEM images of Pd^{II}-LUZ1 (a), and Pd@NHCS(500) (b).

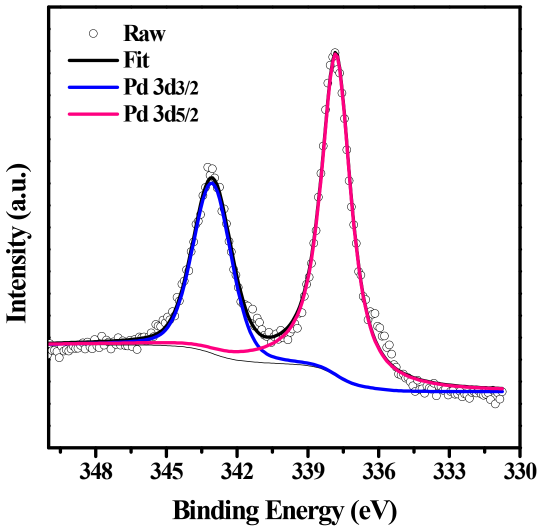

Figure S2. XPS spectrum of the Pd 3d region for Pd^{II}-LUZ1.

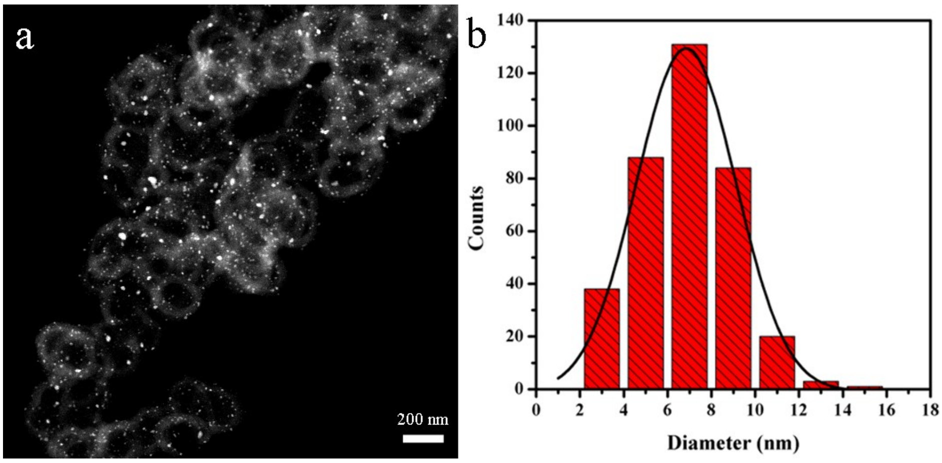

Figure S3. DFSTEM image of Pd@NHCS(500) (a) and the corresponding size distribution of Pd NPs (b).

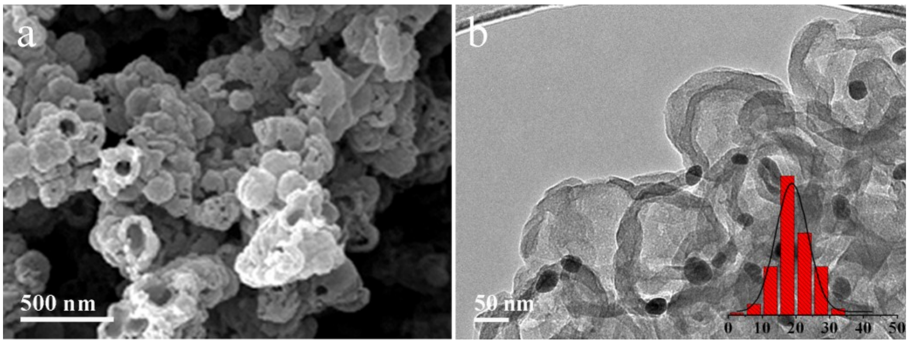

Figue S4. (a) FESEM image of Pd@NHCS(600). (b) TEM image of Pd@NHCS(600) and the corresponding size distribution of Pd NPs (inset).

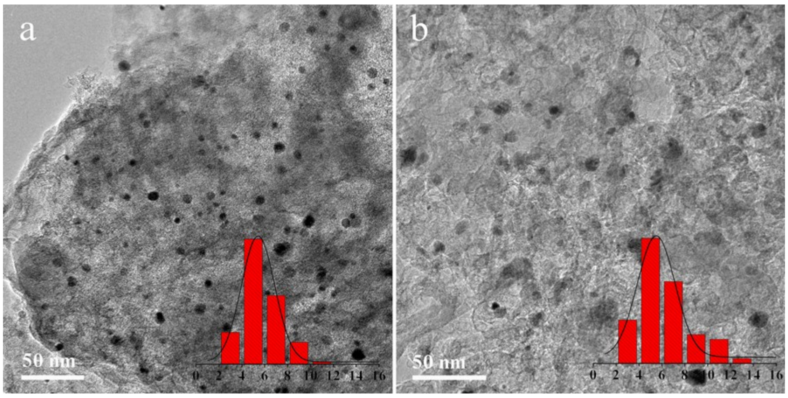

Figure S5. TEM images of Pd/N-C(500) (a) and Pd/C(500) (b). Insets in (a) and (b) show the particle size distributions of Pd NPs.

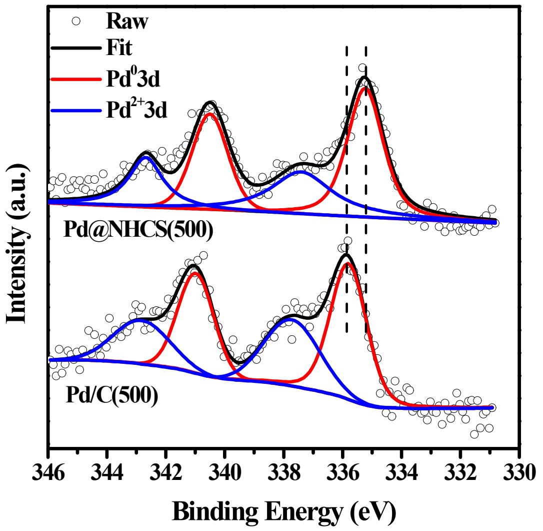

Figure S6. XPS spectra of the Pd 3d region for Pd@NHCS(500) and Pd/C(500).

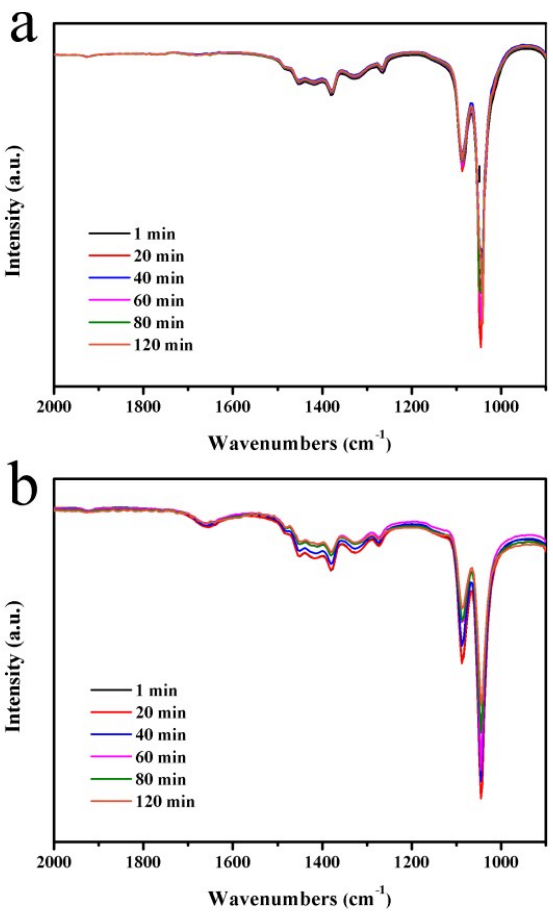

Figure S7. ATR-IR difference spectra of N_2-saturated ethanol solutions on (a) Pd@NHCS(500) (spectrum of the NHCS(500) film was subtracted), and (b) Pd/C(500) (spectrum of the Pd/C(500) film was subtracted).

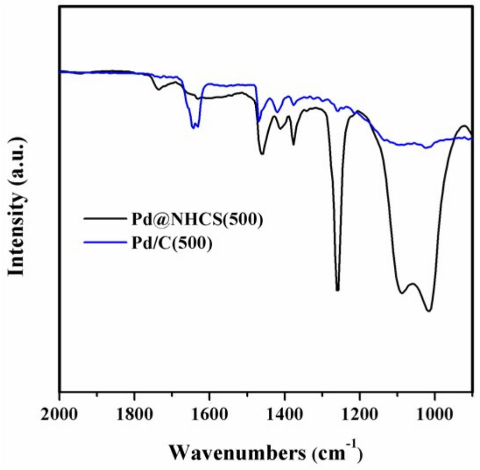

Figure S8. ATR-IR spectra of Pd@NHCS(500) and Pd/C(500).

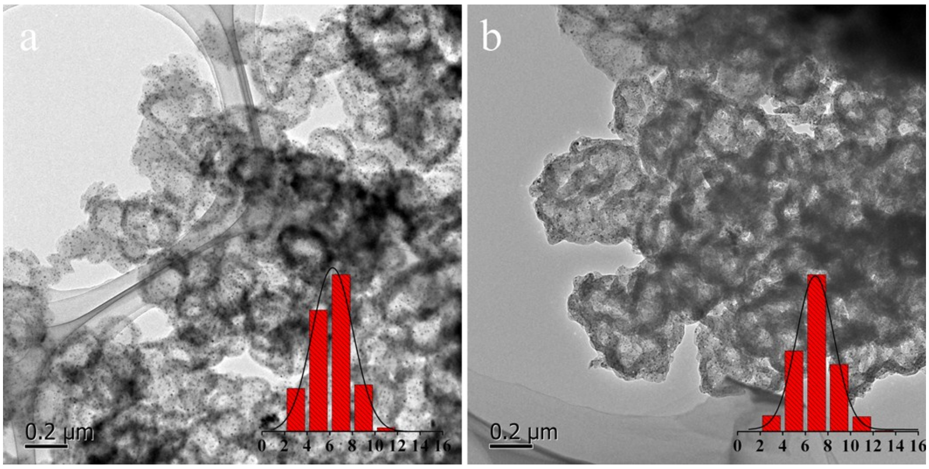

Figure S9. TEM image of 3.6% Pd/N-C(500) (a) and 5.0% Pd/N-C(500) (b). Insets in (a) and (b) show the particle size distributions of Pd NPs.

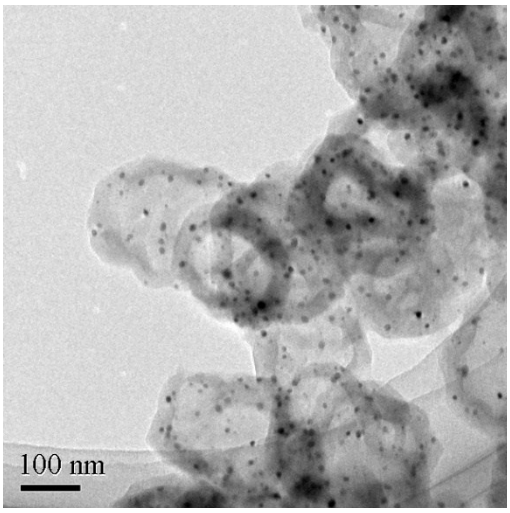

Figure S10. TEM image of Pd@NHCS(500) after being reused for five times.

Table S1. Characterization results of the materials.

Sample	C content[a] (wt%)	H content[a] (wt%)	Pd content[b] (wt%)	N content[a] (wt%)
LZU1	81.0	5.0	--	14.0
Pd@C-N-500	71.8	3.1	2.4	11.0
Pd@C-N-600	69.7	2.7	3.6	8.6

[a] Measured by elemental analysis.

[b] Measured by AAS.

Table S2. Surface areas and pore volumes of the Pd@NHCS(500) and Pd@NHCS(600).

Sample	S_{BET} [$m^2\ g^{-1}$]	$S_{Langmuir}$ [$m^2\ g^{-1}$]	V_{pore} [$cm^3\ g^{-1}$]
Pd@NHCS(500)	468	597	0.19
Pd@NHCS(600)	527	669	0.24

Table S3. Results of hydrogenation of nitrobenzene.[a]

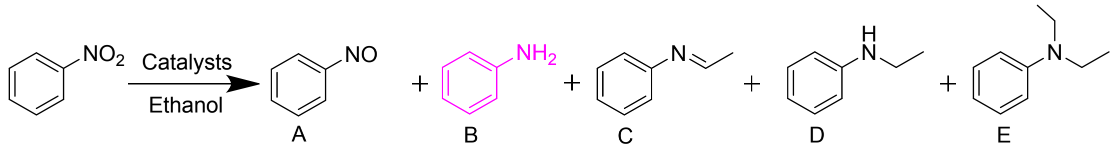

Entry	Catalyst	Time [min]	Conversion [%][b]	Selectivity [%][b]				
				A	B	C	D	E
1	Pd@NHCS(500)	50	>99	0.3	88.0	3.5	8.2	--
2	Pd@NHCS(600)	100	>99	1.0	88.0	0.2	10.8	--
3	Pd/N-C(500)	120	>99	0.4	88.0	4.1	7.5	--
4	Pd/C(500)	120	89	1.8	12.0	6.2	68.6	11.4

[a] Reaction condition: nitrobenzene (0.1 mmol), Pd catalyst (1 mol% Pd), ethanol (2 mL), 1 atm H_2, 25 °C. [b] The conversion and selectivity was determined by GC-MS analysis.

Table S4. Results of oxidation of cinnamyl alcohol.[a]

Entry	Catalyst	Time (h)	Yield (%)[b]
1	Pd@NHCS(500)	10	>99
2	Pd/C-N(500)	10	60
3	Pd/C(500)	10	24

[a] Reaction condition: cinnamyl alcohol (0.1 mmol), Pd catalyst (Pd 1 mol%), toluene (2 ml), 80 °C, under air. [b] The yield was determined by GC-MS analysis.

Appendix The MS spectra for the products listed in Figure 4 and Tables S3.

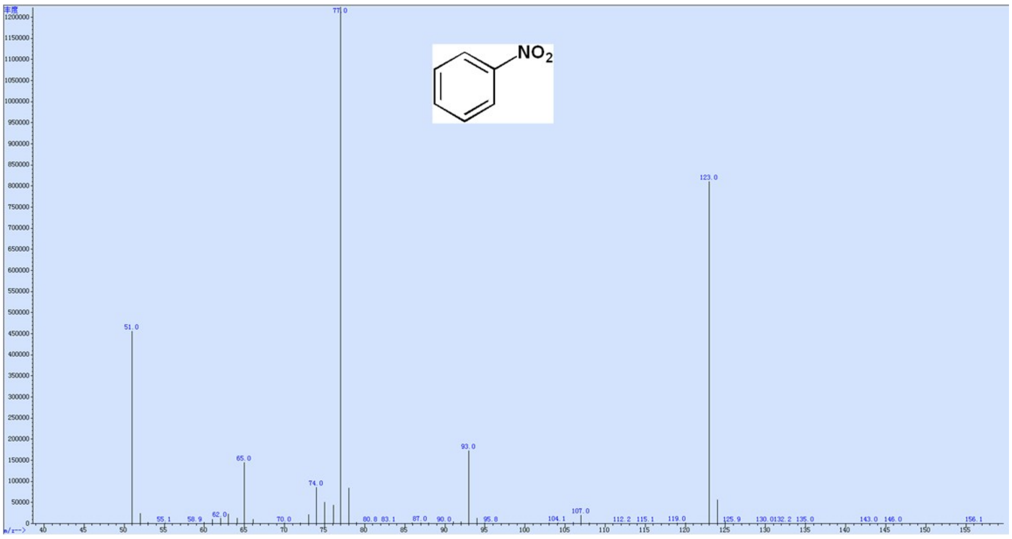

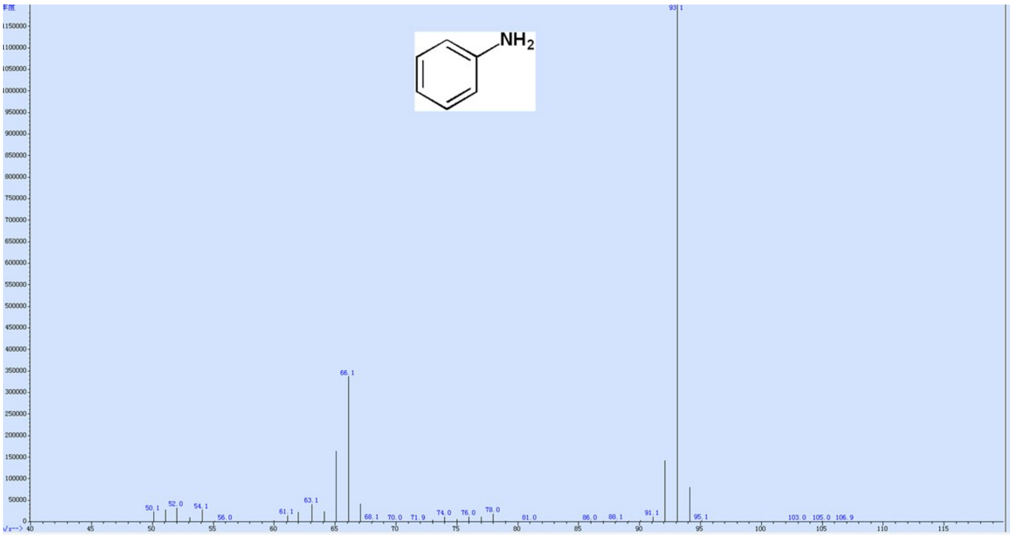

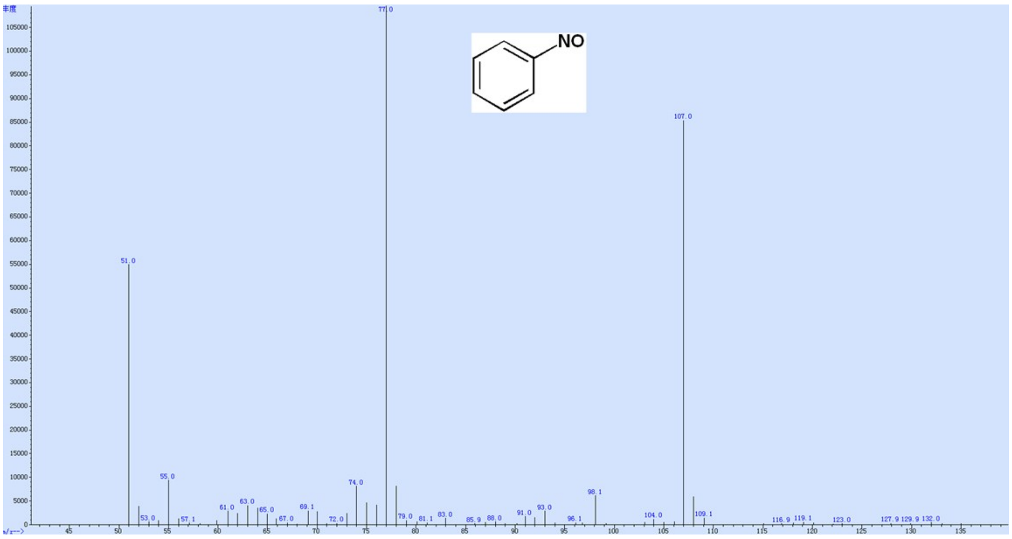
NO
77.0
107.0
51.0
55.0
74.0
98.1

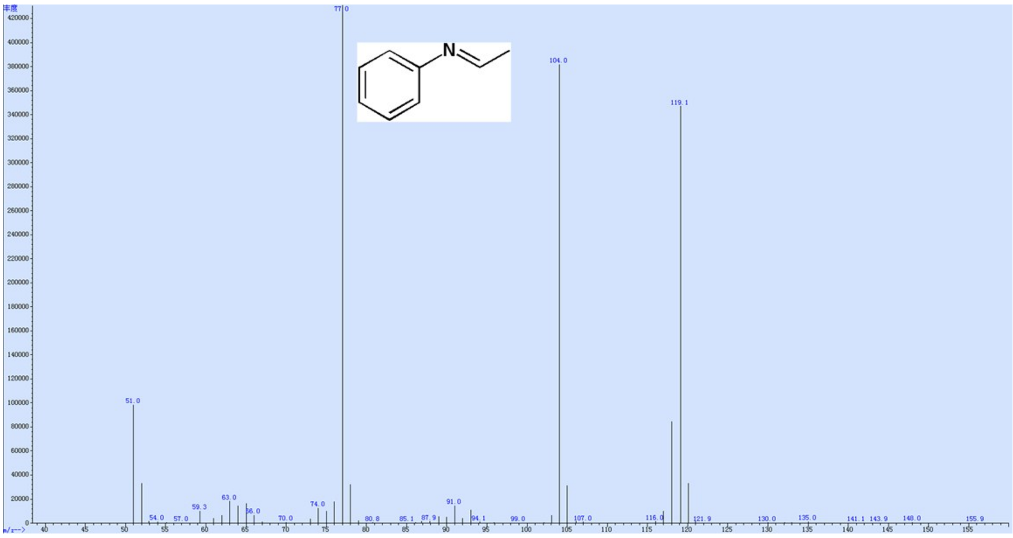
N
77.0
104.0
119.1
51.0
63.0
91.0

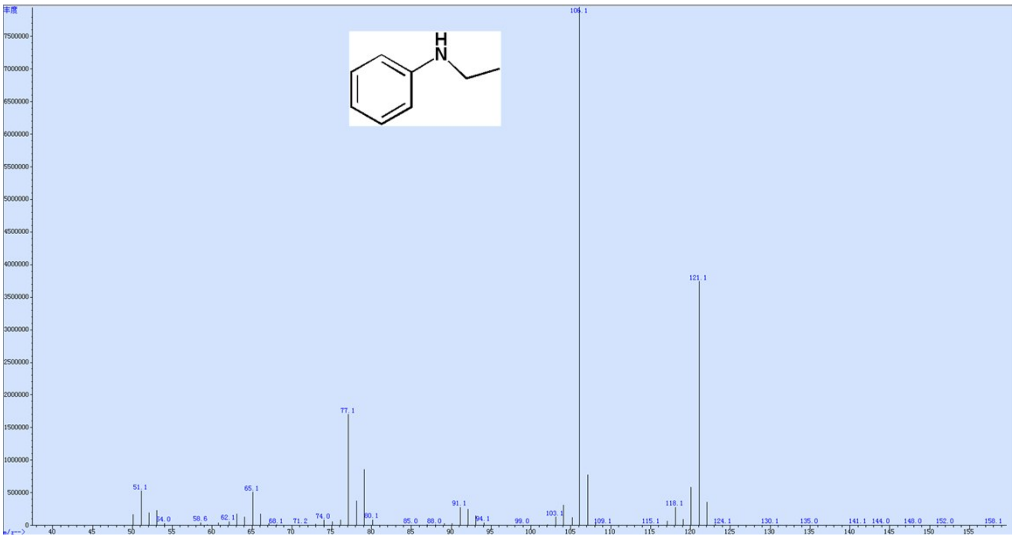
H
N
106.1
121.1
77.1
51.1
65.1
118.1

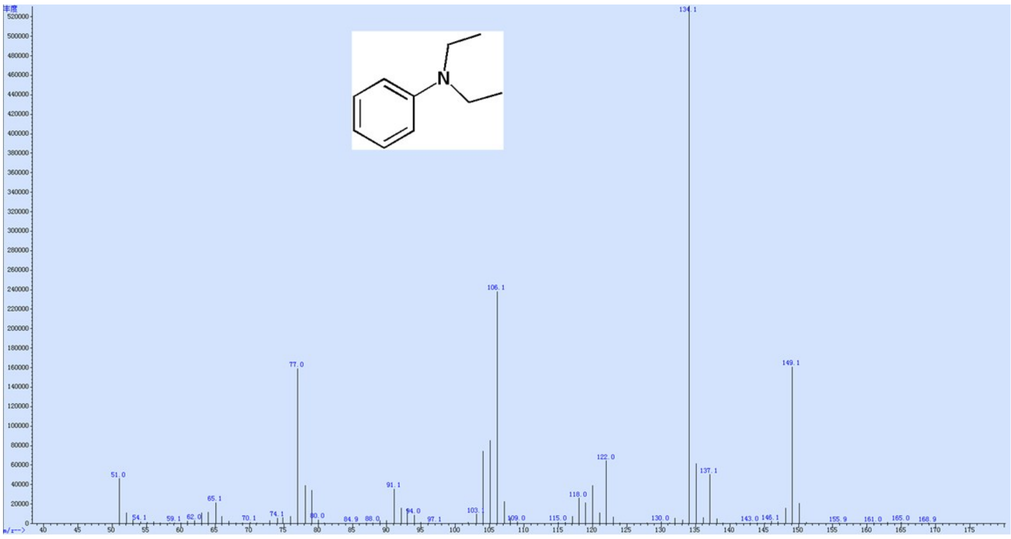

丰度
m/z-->
N
134.1
106.1
77.0
149.1
122.0
137.1
118.0
91.1
51.0
65.1
